# Temporal stability of *Glossina fuscipes fuscipes *populations in Uganda

**DOI:** 10.1186/1756-3305-4-19

**Published:** 2011-02-14

**Authors:** Richard Echodu, Jon S Beadell, Loyce M Okedi, Chaz Hyseni, Serap Aksoy, Adalgisa Caccone

**Affiliations:** 1 Faculty of Science, Gulu University, Uganda; 2 Department of Ecology and Evolutionary Biology, Yale University, New Haven, Connecticut, USA; 3 National Livestock Resources Research Institute, Tororo, Uganda; 4 Yale School of Public Health, Yale University, New Haven, Connecticut, USA

## Abstract

**Background:**

*Glossina fuscipes*, a riverine species of tsetse, is the major vector of human African trypanosomiasis (HAT) in sub-Saharan Africa. Understanding the population dynamics, and specifically the temporal stability, of *G. fuscipes *will be important for informing vector control activities. We evaluated genetic changes over time in seven populations of the subspecies *G. f. fuscipes *distributed across southeastern Uganda, including a zone of contact between two historically isolated lineages. A total of 667 tsetse flies were genotyped at 16 microsatellite loci and at one mitochondrial locus.

**Results:**

Results of an AMOVA indicated that time of sampling did not explain a significant proportion of the variance in allele frequencies observed across all samples. Estimates of differentiation between samples from a single population ranged from approximately 0 to 0.019, using Jost's D_EST_. Effective population size estimates using momentum-based and likelihood methods were generally large. We observed significant change in mitochondrial haplotype frequencies in just one population, located along the zone of contact. The change in haplotypes was not accompanied by changes in microsatellite frequencies, raising the possibility of asymmetric mating compatibility in this zone.

**Conclusion:**

Our results suggest that populations of *G. f. fuscipes *were stable over the 8-12 generations studied. Future studies should aim to reconcile these data with observed seasonal fluctuations in the apparent density of tsetse.

## Introduction

Tsetse flies, *Glossina *spp (Diptera: Glossinidae) transmit several species of pathogenic trypanosomes causing Human African Trypanosomiasis (HAT) and African Animal Trypanosomiasis (AAT). HAT affects human welfare directly through the chronic and acute forms of the disease caused by *Trypanosoma brucei gambiense *and *T. b. rhodesiense *respectively. AAT, on the other hand, stands as a major obstacle to the development of more efficient and sustainable livestock production systems in tsetse-infested areas [1]. A major challenge to controlling HAT is lack of suitable prophylactic drugs and vaccines against trypanosomiasis. Furthermore, chemotherapeutic agents for treatment of HAT are expensive, difficult to administer in remote areas and exhibit poor safety profiles. Consequently, vector control remains a viable alternative for large-scale control of trypanosomiasis.

Understanding tsetse population dynamics is critical for determining which control strategy is most appropriate (e.g., suppression, eradication), for choosing the best method for enacting that strategy (e.g., traps, insecticide-treated cattle, sterile insect technique), and for determining the scale at which vector control activities must be implemented [2]. Determinants of population dynamics include both life history and ecological correlates such as mating system, dispersal ability and population size, which influence the extent to which tsetse populations can recover from refugia following intervention, or re-colonize a cleared zone from neighboring sources. Recently, the use of population genetics has provided insights into tsetse ecology [3], with important ramifications for the implementation of control programs [4]. For example, studies of tsetse in Guinea and Senegal have identified populations that are sufficiently isolated to warrant attempts at complete elimination [5-7]. Elsewhere though, studies have documented relatively high levels of gene flow, necessitating integration of barriers into elimination schemes [8] or warranting an area-wide control effort that encompasses the dispersal-linked populations [9,10].

Across Africa, *Glossina fuscipes *is one of the most important vectors of HAT, transmitting an estimated 90% of all disease cases [11]. *Glossina fuscipes *is a member of the *palpalis *group of tsetse, which inhabit low bushes or forests at the margins of rivers, lakes or temporarily-flooded scrub land. In eastern Africa, populations of the subspecies *G. f. fuscipes *appear to respond to seasonal weather patterns, often disappearing during the bi-annual dry season from sites where they were previously abundant [12]. If populations in refugia are small, then seasonal bottlenecks could result in large temporal changes in gene frequencies. In order to investigate the impact of seasonal climate changes on population size and to gain further insight into the population dynamics of *G. f. fuscipes*, we evaluated temporal changes in gene frequencies at one mitochondrial locus and 16 microsatellite loci in multiple Ugandan populations. Our sampling scheme included three populations situated at a zone of contact between two divergent lineages of *G. f. fuscipes*. These two lineages exhibit distinct mitochondrial DNA (mtDNA) haplotypes and strong differentiation at microsatellite loci, suggesting a long history of isolation, and providing a unique opportunity to monitor their interaction over time [9,10].

## Materials and methods

### Tsetse collection and study area

Tsetse flies were collected using biconical traps [13] during the period from March 2008 to January 2010. All sites were sampled in 2008 [10] and then at least one year later in 2009 or 2010. Four sites were also sampled a third time (Table 1). Each fly was stored individually in 80% ethanol.

**Table 1:** Indices of molecular diversity at mitochondrial and microsatellite loci for temporal samples of *G. f. fuscipes*.

		Microsatellites				Mitochondrial DNA			
			
Sample	Date of Sampling	N	Allelic Richness	H_o_	H_e_	N	No. haplotypes	Haplotype diversity	Nucleotide diversity
BN - 0	March 2008	32	4.2	0.529	0.578	15	3	0.648	0.00538
BN - 8	March 2009	40	3.9	0.568	0.609				
BN - 12	October 2009	64	4.1	0.549	0.574	16	4	0.692	0.00466
BU - 0	March 2008	39	3.5	0.459	0.485	17	1	0.000	0.00000
BU - 8	March 2009	40	3.4	0.476	0.485				
BU - 12	October 2009	40	3.4	0.464	0.477	19	1	0.000	0.00000
JN - 0	March 2008	40	3.2	0.479	0.489	19	3	0.444	0.00731
JN - 13	January 2010	18	3.1	0.460	0.485	18	1	0.000	0.00000
MK - 0	March 2008	40	2.9	0.487	0.460	21	2	0.495	0.00093
MK - 8	March 2009	24	3.0	0.455	0.431				
MK - 12	November 2009	22	3.1	0.418	0.445	21	2	0.467	0.00088
MS - 0	March 2008	40	3.7	0.568	0.547	18	2	0.471	0.00886
MS - 13	January 2010	17	4.4	0.562	0.597	17	3	0.559	0.00964
OK - 0	March 2008	39	3.3	0.452	0.507	17	3	0.471	0.00094
OK - 8	March 2009	40	3.4	0.563	0.546				
OK - 12	October 2009	39	3.4	0.547	0.552	18	2	0.294	0.00055
OT - 0	July 2008	53	4.0	0.508	0.535	20	3	0.426	0.00122
OT - 11	November 2009	40	3.7	0.514	0.540	20	4	0.537	0.00131

**Figure 1:**
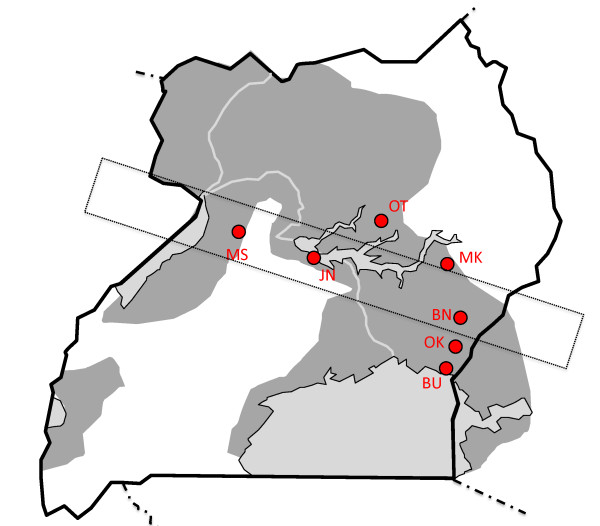
**Map of sites at which populations of *G. f. fuscipes *were sampled in Uganda**. Location codes are shown in Table 1. The dotted line indicates the approximate extent of a zone of contact between two historically isolated groups of tsetse.

Tsetse collections were conducted at seven sites spanning central and southeastern Uganda (Figure 1). These sites generally reflected the riverine/woodland habitat preferred by *G. fuscipes*, but varied somewhat in regard to the immediate environment. Sites at Busime (BU) and Junda (JN) were located in a transition zone between marsh and woodland on the edge of Lake Victoria and Lake Kyoga, respectively. Sites at Bunghazi (BN), Dokolo/Otuboi (OT) and Okame (OK) were situated along permanent streams in a region of mixed agriculture and pastureland. Sampling at Mukongoro (MK) was conducted at the margin of ephemeral wetlands associated with rice cultivation. Sampling at Masindi (MS) was conducted within a region of banana and sugar cane plantations.

The study sites spanned a zone of contact between two divergent groups of *G. fuscipes *co-occuring in Uganda [9,10]. Sites MK and OT were situated north of the zone of contact and flies here were expected to possess solely northern mtDNA haplotypes. Sites BN, JN, and MS were located at the zone of contact and flies here were expected to possess both northern and southern haplotypes. Sites BU and OK were located south of the zone of contact and flies at these sites were expected to possess exclusively southern mtDNA haplotypes.

### DNA Extraction

DNA was extracted from tsetse legs using NucleoSpin 96 Tissue Kits (Clontech, Mountain View, CA) or DNeasy kits (Qiagen, Valencia, CA) following the manufacturer's protocols.

### Mitochondrial DNA sequencing

PCR was used to amplify a 570 bp fragment of mtDNA from a random subset of flies from each population using the primers COIF1 (CCT CAA CAC TTT TTA GGT TTA G) and COIIR1 (GGT TCT CTA ATT TCA TCA AGT A) as described by [10]. We amplified COIF1/COIIR1 in a 25 μl PCR reaction containing 1 × buffer (GoTaq colorless, Promega), 0.8 mM each dNTP, 0.4 mM primers, 1.5 mM MgCl_2 _and 0.5 U Go Taq polymerase. The amplification involved a denaturation step at 95°C for 8 min, followed by 50 cycles each at 94°C for 30 s, 51°C for 30 s, 72°C for 45 s, with a final extension step at 72°C for 7 min. PCR products were sequenced using an ABI Model 3730 automated sequencer (Applied Biosystems, Foster City, CA, USA). Electropherograms were visually inspected and sequences were trimmed to remove poor quality data. The resulting sequences (530 bp) were aligned by eye using the computer program Sequencher 4.2.2 (Gene Codes Corporation).

### Microsatellite genotyping

We genotyped individual flies at 16 loci. We used 11 of the 13 loci described by [10], excluding D05 and Pgp17 due to possible null allele problems. We also employed five new dinucleotide loci identified in the *G. morsitans *genome and optimized for use in *G. fuscipes*: GmmA06, GmmB20, GmmD15, GmmL03, GmmL11 [14]. Amplifications were performed with fluorescently labeled forward primers (6-FAM, HEX and NED) using a touchdown PCR (10 cycles of annealing at progressively lower temperatures from 60°C to 51°C followed by 35 cycles at 50°C) in 12.5 μl reaction volumes employing 1 × buffer, 0.8 mM dNTPs, 2.0 mM MgCl_2 _and 0.5 U Go Taq polymerase. PCR products were multiplexed in groups of two or three and then genotyped on the ABI 3730 automated sequencer. Alleles were scored using the program Genemarker v 5.0 (SoftGenetics) with manual editing of the automatically scored peaks.

### Marker validation and genetic diversity

Microsatellite loci were evaluated for Hardy Weinberg equilibrium (HWE) and linkage disequilibrium (LD) using Genepop version 4.0 [15]. Markov chain parameters were set at 10,000 dememorizations, 1000 batches, 10,000 iterations per batch for HWE and 100,000 dememorizations,1000 batches,10,000 iterations per batch for LD. We used the method of [16] as implemented in MultiTest v.1.2 to correct for multiple tests. Locus- and population-specific estimates of microsatellite allele frequencies were generated using the program Genalex version 6.3 [17]. We used the program FSTAT version 3.1 [18] to calculate allelic richness and the program Arlequin v. 3.5 [19] to calculate observed (H_o_) and expected (H_e_) heterozygosity for each population. DnaSP version 5.0 [20] was used to calculate mtDNA haplotype diversity (H_d_) and nucleotide diversity (π).

### Temporal genetic differentiation and population stability

For microsatellite data, we used Jost's D_EST _[21] to quantify genetic differentiation between populations and between temporal samples from the same population. D_EST _provides a less-biased estimate of differentiation than F_ST _and related statistics, especially when estimated using highly polymorphic microsatellite loci [22]. Locus-specific calculations of D_EST _were performed using the web-based program SMOGD [23] and then averaged across loci. For mtDNA data, we used Fisher's exact test and the statistical software SAS version 9.1 to test for differences in haplotype frequencies among temporal samples from the same population. For both microsatellite and mtDNA data, we performed an analysis of molecular variance (AMOVA) as implemented in Arlequin v. 3.5 [19] to characterize the proportion of the variance in microsatellite allele frequencies or haplotype frequencies that was attributable to differences in date of sampling. For this analysis, we used only the two samples from each population that were separated by the longest time interval.

We estimated current effective population sizes based on temporal changes in microsatellite allele at all seven sites. The effective size of a population (N_e_) is defined as the size of an ideal population (i.e., one of constant size, discrete generations, and negligible selection and gene flow), which would exhibit the same genetic characteristics as the population at hand [24]. N_e_, therefore, reflects the rate of change in gene frequencies due to random drift alone [25]. We used two methods: the moment-based approach [26] and a likelihood approach implemented in TM3 [27]. Estimates were generated using the software NeEstimator [28]. For TM3, we employed 100,000 updates and a maximum N_e _of 20,000.

For all analyses, we assumed that *G. fuscipes *undergoes approximately 8 generations per year using observations from colony flies (~7.3 generations per year, [29] ~8.5 generations per year at 25°C, [30] and those reported in other studies of the palpalis group (*G. palpalis gambiensis *and *G. palpalis palpalis*) in Guinea and Equatorial Guinea [5,31]. All populations were evaluated at an interval of at least one year (~8 generations apart). For four populations (BU, OK, BN, MK), we generated estimates at two different sampling intervals (0 to 8 generations, and 0 to 12 generations).

For each temporal sample in all seven populations, we also tested for an excess of heterozygosity relative to observed allelic diversity, which may be indicative of a recent bottleneck [32]. For each temporal sample, tests of heterozygosity excess were performed separately for each microsatellite locus. Significance was assessed across loci using Wilcoxon's test, which is the most appropriate test given the number of microsatellite loci evaluated. All tests were performed using the program BOTTLENECK [33].

## Results

### Marker validation and diversity

We genotyped a total of 667 tsetse flies at 16 microsatellite loci. We detected 17 values of F_IS _(out of 288) that exhibited significant departures from HWE at p < 0.05 (Additional file 1: Table S1). Assessed by locus, the number of significant F_IS _values observed was consistent with chance at an overall value of p < 0.05. Assessed by population, the number of significant F_IS _values observed was consistent with chance for all populations except the sample representing generation 12 from BN. Following sequential Bonferroni correction, only one locus pair exhibited significant linkage, and only in one population, confirming previous work showing that these loci were unlinked [10,14].

Microsatellite diversity was lowest in the samples from Mukongoro (MK) and highest in the samples from Bunghazi (BN). Allelic richness ranged from 3.0 to 4.4 and expected heterozygosity (H_E_) ranged from 0.418 to 0.609, (Table 1). MtDNA haplotype diversity was relatively low across samples with the number of haplotypes ranging from 1 to 4. As expected, nucleotide diversity was generally higher in populations from the zone of contact which were composed of flies with both northern and southern ancestry. We detected only two haplotypes that had not been previously reported [10]. Both of these haplotypes were recovered in population OT and differed by just one substitution from Hap26 or Hap27 [10].

### Temporal variation in genetic diversity

**Figure 2:**
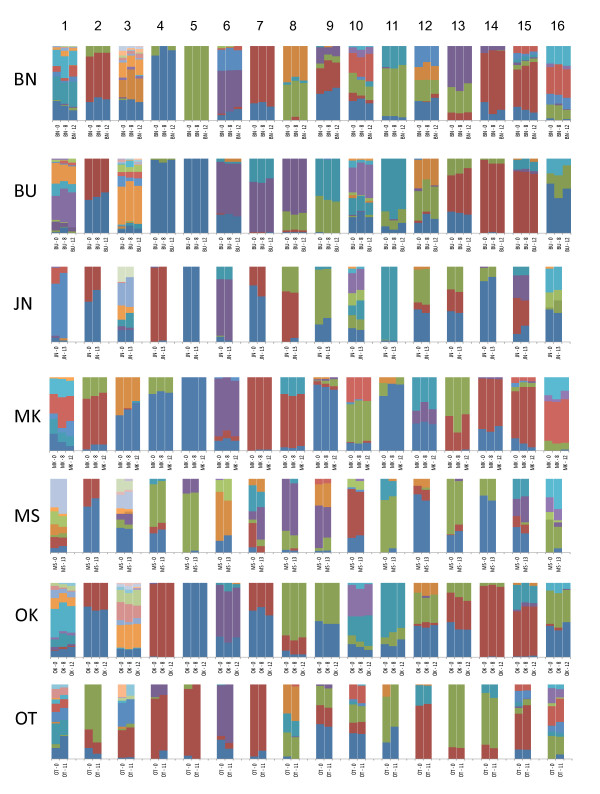
**Microsatellite allele frequencies observed in seven populations of *G. f. fuscipes *sampled at either two or three different times**. Numbers after location code indicate the time interval (in generations) since the first sampling.

Variation in allele frequencies by population and locus are depicted in Figure 2. Genetic differentiation between samples taken from the same population but at different times was extremely low, and uniformly lower than the differentiation observed between populations. D_EST _averaged 0.001 for temporally-spaced samples within populations, compared to 0.308 between populations (Additional file 2: Table S2). The largest values of D_EST _among temporally-spaced samples were observed in Masindi (MS generation 0 vs.13, D_EST _= 0.019 ± 0.022) and Otuboi (OT generation 0 vs. 11, D_EST _= 0.013 ± 0.007).

**Figure 3:**
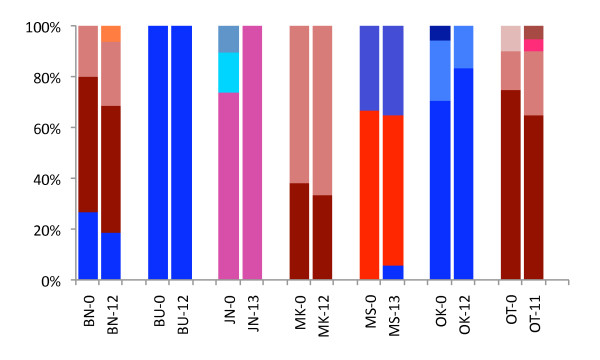
**Mitochondrial haplotype frequencies observed in seven populations of *G. f. fuscipes *sampled at two different time periods**. Numbers after location codes indicate the time interval (in generations) since the first sampling. Only temporal samples from Junda (JN) differed significantly.

Mitochondrial haplotype frequencies also exhibited little change over time (Figure 3). We observed a significant change in haplotype frequencies only between the two temporally spaced samples from Junda (JN, p = 0.046). This was attributable to the loss of the two least common haplotypes in the sample representing generation 13.

An analysis of molecular variance using both microsatellite allele frequencies and haplotype frequencies suggested that differences between temporally-spaced samples explained an insignificant amount of the overall genetic variation (Table 2). Differences among sites, on the other hand, contributed significantly to the overall variation in genetic diversity. The percent variation explained was greater in the case of mtDNA data, compared to microsatellite data.

**Table 2:** Results of an AMOVA testing for temporal genetic structure in seven populations of *G. fuscipes *sampled in 2008 and also in 2009/2010.

	d.f	Sum of squares	Variance components	% Variation	p
**mtDNA 2008 vs. 2009/2010**					
Among temporal groups	1	0.4	-0.24319	-11.1	0.997
Among sites within groups	12	374.6	1.66812	76.4	0
Within sites	242	183.3	0.75752	35.7	0
**microsatellites 2008 vs. 2009/2010**					
Among temporal groups	1	11.6	-0.15553	-3.2	1
Among sites within groups	12	986.2	1.07307	22.0	0
Within sites	1022	4057.4	3.96592	81.2	0

Results for microsatellites represent the weighted average over 16 loci. Variance components for which the expected value is zero may be slightly negative by chance.

### Effective size

Estimates of N_e _were generated for the seven populations based on microsatellite allele frequency changes observed among samples collected at different times from the same population. Momentum-based estimates ranged from 216 to infinity, but only the estimate from OT was bounded by a 95% confidence interval that did not include infinity (Table 3). Likelihood estimates ranged from 152 to 19,550 and all estimates were bounded by 95% confidence intervals that included 20,000, the maximum value of N_e _considered (Table 3).

**Table 3:** Effective population size (N_e_) and 95% confidence intervals (CI) for *G. f. fuscipes *populations.

Population	Interval sampled (generations)	N_e _- moment	95% CI	N_e _- likelihood	95% CI
BN	12	91926	575-infinity	13776	404-20,000
	8	1774	257.8-infinity	922.1	0-20,000
BU	12	7144	452-infinity	1203	0-20,000
	8	711	200-infinity	852	0-20,000
JN	13	infinity	293-infinity	2118	0-20,000
MK	12	1061	194-infinity	8024	393-20,000
	8	2170	161-infinity	11791	128-20,000
MS	13	443	154-infinity	19550	460-20,000
OK	12	infinity	807-infinity	19139	878-20,000
	8	infinity	378-infinity	18098	425-20,000
OT	11	216	117-439	152	95-20,000

Ne was calculated using the moments based temporal method of Waples (1989) and the likelihood approach of Berthier et al. (2002). For four populations, Ne was calculated using samples collected at intervals of both 8 and 12 generations from the initial sampling.

For populations MK and OK, estimates of N_e _were similar regardless of whether the calculations were performed using data for generations 0 and 8 or generations 0 and 12. In populations BN and BU, however, estimates of N_e _derived from the moment method differed by an order of magnitude depending on whether the sample representing 8 generations or 12 generations was included. In population BN, the estimate of N_e _generated by the Likelihood method was similarly unstable.

### Population bottlenecks

Following Bonferonni correction, seven samples (taken from Bunghazi (BN), Masindi (MS), Okame (OK) and Otuboi (OT)) exhibited significant signatures of a recent population bottleneck under the infinite allele model (IAM) model. Only one of these samples (MS generation 0) also tested positive for a bottleneck under the two phase model (TPM; Table 4). Samples from Busime (BU) and Mukongoro (MK) exhibited the least evidence for past bottlenecks (all but one p-value >> 0.05), however power to detect a bottleneck may have been low in MK on account of relatively low genetic diversity (Table 1).

**Table 4:** Significance of tests for population bottlenecks assessed using a Wilcoxon test under an infinite allele (IAM) or two-phase (TPM) model of microsatellite evolution.

Sample	p (IAM)	p (TPM)
BN - 0	**0.00067**	0.07193
BN - 8	**0.00031**	0.09686
BN - 12	**0.00038**	0.10388
BU - 0	0.22714	0.66061
BU - 8	0.17957	0.73776
BU - 12	0.07571	0.51102
JN - 0	0.02063	0.12619
JN - 13	0.06027	0.1514
MK - 0	0.0365	0.2106
MK - 8	0.16513	0.31934
MK - 12	0.2106	0.53296
MS - 0	**0.00001**	**0.00258**
MS - 13	0.01248	0.39098
OK - 0	0.01077	0.0535
OK - 8	**0.00168**	0.02094
OK - 12	**0.00043**	0.02899
OT - 0	0.00655	0.20187
OT - 11	**0.00002**	0.02396

Values in bold remained significant after Bonferroni correction (p < 0.0028).

## Discussion

We assessed changes in genetic composition of seven tsetse populations in southeast Uganda in order to gain insight into the population dynamics of *G. f. fuscipes*. In general, our results provide evidence for temporal stability of *G. f. fuscipes *populations over the one to two year period that we examined. With the exception of just one or two populations discussed below, mitochondrial haplotype frequencies and microsatellite allele frequencies exhibited little change over time and effective population sizes were generally large.

Compared to other riverine species of tsetse, estimates of N_e _for *G. f. fuscipes *were similar to or larger than estimates for *G. palpalis palpalis *in Equatorial Guinea [31] and 2 to 3 orders of magnitudes larger than estimates for *G. p. gambiensis *on islands off the coast of Guinea [5]. Values of N_e _for *G. f. fuscipes *populations were also generally larger than estimates for a savannah species, *G. pallidipes*, in Kenya [34]. The large effective population sizes and overall stability of *G. f. fuscipes *populations support the hypothesis [35] that seasonal variation in tsetse numbers, in which larva develop in utero, should be relatively small, since they do not depend on surface water or moist media for breeding. Nonetheless, the lack of variation in genetic structure over time is surprising given the reduced abundance of *G. f. fuscipes *observed during the dry season in Uganda [12]. To reconcile our results with this observation, which may reflect the low efficiency of traps used for monitoring [36,37], we suggest that populations of *G. f. fuscipes *in dry season refugia remain large, and that seasonal invasion of marginal wet-season habitat (e.g., at Mukongoro, Bunghazi) must occur in waves of tsetse that are large enough to be representative of the refugia population. Large populations of pupa, which develop in the ground over a period of weeks, may also help to ensure the continuity of tsetse populations and would contribute to reducing the variance in genetic changes over time.

In contrast to the other populations, estimates of N_e _were low for populations MS and especially OT, where both moment and likelihood methods produced values of only about 200. These values could be indicative of small populations. N_e _may also be influenced by overlapping generations and temporal variance in reproductive success as well as the forces of selection, mutation and migration. In this study, however, the low values of N_e _observed in these populations probably reflected small differences in the location of trapping sites used for the two temporal samples. Generation 13 from MS was sampled at a distance of about 4 km from the original site at which generation 0 was sampled. Likewise, generation 11 from OT was sampled at a single site that was 11-20 km from the relatively widely dispersed sites from which generation 0 was sampled. Thus, for these sites, which were the only two sites sampled at different locations across years, fine-scale spatial genetic variation could be responsible for the apparent temporal variation in gene frequencies, thus depressing estimates of N_e_.

Given that genetic variation in MS and OT samples can probably be attributed to microgeographic variation, the change in genetic composition of the population at JN likely reflects the only significant temporal change observed in this study. Although microsatellite allele frequencies were largely invariate, mtDNA haplotype frequencies here differed significantly between generation 0 and generation 13. Junda (JN), along with sites BN and MS, lies along a narrow zone of contact between two long-diverged and historically-isolated groups of *G. f. fuscipes *[9,10]. In 2008, populations at all three of these sites harbored both "southern" and "northern" mtDNA haplotypes. Interestingly, in Junda, individuals with the "southern" haplotypes disappeared from the sample after 13 generations. This could be due to a particularly small population of females and stochastic variation in female reproductive success, although in tsetse, the latter is more likely to be true among males than females [5]. Mating success can also be influenced by *Wolbachia*, a symbiont that may impose mating barriers due to cytoplasmic incompatibility between infected and uninfected tsetse individuals [38], thereby biasing mating in favor of infected females and potentially producing mitochondrial sweeps [39]. Given the change in mtDNA observed at Junda, flies here should be examined for *Wolbachia*. If present, the zone of contact in Uganda may provide a unique opportunity to monitor symbiont-induced population changes over time.

## Competing interests

The authors declare that they have no competing interests.

## Authors' contributions

RE and JSB contributed to field collections, performed lab work, analyzed the data, and drafted an initial version of the manuscript. CH also performed lab work and data analysis. LMO, SA and AC helped design the study, coordinated fieldwork and provided guidance on the manuscript. All authors read and approved the final manuscript.

## Supplementary Material

Additional file 1 **Table S1**. F_IS _values for the 16 microsatellite loci. Significance was assessed at p < 0.05 (*) and a Bonferroni-corrected value of p < 0.0028 (bold). Low variability precluded calculation of F_IS _in some populations (n/a). Click here for file

Additional file 2 **Table S2**. Pairwise estimates of genetic differentiation (Jost's D_EST_) between samples taken from seven populations of *G. f. fuscipes*. Estimates of differentiation (below diagonal) and associated standard error (above diagonal) between populations of flies sampled at the same site but different times are shaded in grey. Click here for file

## References

[B1] Itard J Cuisance D Tacher G Trypanosomoses: historique répartition géographique. Principales maladies infectieuses et parasitaires du bétail Europe et Régions Chaudes. Editions Tec et Doc and Editions Médicales Internationales 2003 2 Lavoisier, Paris 1607 1615

[B2] Torr S Hargrove J Vale G Towards a rational policy for dealing with tsetse Trends Parasitol 2005 21 537 541 10.1016/j.pt.2005.08.021 16140579

[B3] Krafsur ES Tsetse flies: genetics, evolution, and role as vectors Infect Genet Evol 2009 9 124 141 10.1016/j.meegid.2008.09.010 18992846 PMC2652644

[B4] Solano P Ravel S De Meeus T How can tsetse population genetics contribute to African trypanosomiasis control? Trends Parasitol 2010 26 5 255 263 10.1016/j.pt.2010.02.006 20202905

[B5] Solano P Ravel S Bouyer J Camara M Kagbadouno MS Dyer N Bardes L Herault D Donnelly MJ De Meeus T The Population Structure of *Glossina palpalis gambiensis *from Island and Continental Locations in Coastal Guinea PLoS Negl Trop Dis 2009 3 3 e392 10.1371/journal.pntd.0000392 19290038 PMC2652410

[B6] Kagbadouno M Camara M Bouyer J Hervouet JP Courtin F Jamonneau V Morifaso O Kaba D Solano P Tsetse elimination: its interest and feasibility in the historical sleeping sickness focus of Loos islands, Guinea Parasite 2009 16 29 35 19353949 10.1051/parasite/2009161029

[B7] Solano P Kaba D Ravel S Dyer NA Sall B Vreysen MJ Seck MT Darbyshir H Gardes L Donnelly MJ De Meeûs T Bouyer J Population Genetics as a Tool to Select Tsetse Control Strategies: Suppression or Eradication of *Glossina palpalis gambiensis *in the Niayes of Senegal PLoS Negl Trop Dis 2010 4 5 e692 10.1371/journal.pntd.0000692 20520795 PMC2876113

[B8] Bouyer J Balenghien T Ravel S Vial L Sidibé I Venon ST Solano P Demeeus T Population sizes and dispersal pattern of tsetse flies: rolling on the river Molecular Ecology 2009 18 2787 2797 10.1111/j.1365-294X.2009.04233.x 19457176

[B9] Abila PP Slotman MA Parmakelis A Dion KB Robinson AS Muwanika VB Enyaru JCK Okedi LM Aksoy S Caccone A High levels of genetic differentiation between Ugandan *Glossina fuscipes fuscipes *populations separated by Lake Kyoga PLoS Negl Trop Dis 2008 2 5 e242 10.1371/journal.pntd.0000242 18509474 PMC2386243

[B10] Beadell JS Hyseni C Abila PP Azabo R Enyaru JCK Ouma JO Mohammend YO Okedi LM Aksoy S Caccone A Phylogeography and population structure of *Glossina fuscipes fuscipes *in Uganda: Implications for control of tsetse PLoS Negl Trop Dis 2010 4 3 e636 10.1371/journal.pntd.0000636 20300518 PMC2838784

[B11] Omolo MO Hassanali A Mpiana S Esterhuizen J Lindh J Lehane MJ Solano P Rayaisse JB Vale GA Torr J Tirado I Prospects for Developing Odour Baits To Control *Glossina fuscipes *spp., the Major Vector of Human African Trypanosomiasis PLoS Negl Trop Dis 2009 3 5 e435 10.1371/journal.pntd.0000435 19434232 PMC2674566

[B12] Katunguka-Rwakishaya E Kabagambe EK Tsetse survey in Mukono district, south-east Uganda: population structure, distribution and blood meal status Trop Anim Hlth Prod 1996 28 151 157 8809978

[B13] Challier A Laveissiere C Un nouveau pie'ge pour la capture des glossines (Glossina: Diptera, Muscidae): description et essais sur le terrain Cah ORSTOM sér Ent Méd Parasitol 1973 11 251 262

[B14] Hyseni C Beadell J Gomez Ocampo Z Ouma J Okedi L Gaunt M Caccone A The *Glossina morsitans morsitans *(Diptera: Glossinidae) genome as a source of microsatellite markers for other tsetse fly (Glossina) species Molecular Ecology Resources 2011 11 3

[B15] Rousset F GENEPOP'007: a complete re-implementation of the Genepop software for Windows and Linux Mol Ecol Res 2008 8 103 106 10.1111/j.1471-8286.2007.01931.x 21585727

[B16] De Meeûs T Guegan JF Teriokhin A MultiTest V.1.2, a program to binomially combine independent tests with a comparison to other related methods on proportional data BMC Bioinformatics 2009 10 443 20030807 10.1186/1471-2105-10-443 PMC2811122

[B17] Peakall R Smouse PE GENALEX 6: genetic analysis in Excel. Population genetic software for teaching and research Molecular Ecology Notes 2006 6 288 295 10.1111/j.1471-8286.2005.01155.x PMC3463245 22820204

[B18] Goudet J FSTAT (version 1.2): a computer program to calculate Fstatistics J Heredity 1995 86 485 486

[B19] Excoffier L Laval G Schneider S Arlequin (version 3.0): an integrated software package for population genetics data analysis Evolutionary Bioinformatics 2005 1 47 50 PMC2658868 19325852

[B20] Rozas JJC Sanchez-Delbarrio X Messeguer Rozas R DnaSP, DNA polymorphism analyses by the coalescent and other methods Bioinformatics 2003 19 2496 2497 10.1093/bioinformatics/btg359 14668244

[B21] Jost L G(ST) and its relatives do not measure differentiation Molecular Ecology 2008 17 4015 4026 10.1111/j.1365-294X.2008.03887.x 19238703

[B22] Heller R Siegismund HR Relationship between three measures of genetic differentiation G(ST), D-EST and G'(ST): how wrong have we been? Molecular Ecology 2009 18 2080 2083 10.1111/j.1365-294X.2009.04185.x 19645078

[B23] Crawford NG smogd: software for the measurement of genetic diversity Molecular Ecology Resources 2010 10 556 557 10.1111/j.1755-0998.2009.02801.x 21565057

[B24] Wright S Evolution in Mendelian populations Genetics 1931 16 97 159 17246615 10.1093/genetics/16.2.97 PMC1201091

[B25] Charlesworth D Plant sex determination and sex chromosomes Heredity 2002 88 94 101 10.1038/sj.hdy.6800016 11932767

[B26] Waples RS A generalized approach for estimating effective population size from temporal changes in allele frequency Genetics 1989 121 379 391 2731727 10.1093/genetics/121.2.379 PMC1203625

[B27] Berthier P Beaumont MA Cornuet JM Luikart G Likelihood-based estimation of the effective population size using temporal changes in allele frequencies: a genealogical approach Genetics 2002 160 741 751 11861575 10.1093/genetics/160.2.741 PMC1461962

[B28] Jorde PE Ryman N Unbiased estimator for genetic drift and effective population size Genetics 2007 177 927 935 10.1534/genetics.107.075481 17720927 PMC2034655

[B29] Krafsur ES Marquez JG Ouma JO Structure of some East African *Glossina fuscipes fuscipes *(Diptera: Glossinidae) populations Med Vet Entomol 2008 22 222 227 10.1111/j.1365-2915.2008.00739.x 18816270 PMC2562567

[B30] Hargrove JW Extinction probabilities and times to extinction for populations of tsetse flies *Glossina *spp. (Diptera: Glossinidae) subjected to various control measures Bulletin of Entomological Research 2005 95 13 21 10.1079/BER2004335 15705210

[B31] Dyer NA Furtado A Cano J Ferreira F Afonso MO Mabale NN Asumu PN Lima SC Benito A Weetman D Donnelly MJ Pinto J Evidence for a discrete evolutionary lineage within Equatorial Guinea suggests that the tsetse fly *Glossina palpalis palpalis *exists as a species complex Molecular Ecology 2009 18 3268 3282 10.1111/j.1365-294X.2009.04265.x 19619197

[B32] Cornuet JM Luikart G Description and power analysis of two tests for detecting recent population bottlenecks from allele frequency data Genetics 1996 144 2001 2014 8978083 10.1093/genetics/144.4.2001 PMC1207747

[B33] Piry S Luikart G Cornuet JM BOTTLENECK: A computer program for detecting recent reductions in the effective population size using allele frequency data J Heredity 1999 90 502 503 10.1093/jhered/90.4.502

[B34] Ouma JO Marquez JG Krafsur ES Macrogeographic population structure of the tsetse fly, *Glossina pallidipes *(Diptera: Glossinidae) Bull Entomol Res 2005 95 437 447 10.1079/BER2005376 16197564 PMC1560345

[B35] Hargrove JW Maudlin I, Holmes PH, Miles MA Tsetse population dynamics The Trypanosomiases 2004 CABI Publishing, Wallingford 113 138 full_text

[B36] Bouyer J Seck MT Sall B Ndiaye EY Guerrini L Vreysen MJB Stratified entomological sampling in preparation for an area-wide integrated pest management program: the example of *Glossina palpalis gambiensis *(Diptera: Glossinidae) in the Niayes of Senegal J Med Entomol 2010 47 543 552 10.1603/ME09149 20695269 PMC7027262

[B37] Rayaisse JB Tirados I Kaba D Dewhirst SY Logan JG Diarrassouba A Salou E Omolo MO Solano P Lehane MJ Pickett JA Vale GA Torr SJ Esterhuizen J Prospects for the development of odour baits to control the tsetse flies *Glossina tachinoides *and *G. palpalis s.l* PLOS Negl Trop Dis 2010 4 e632 10.1371/journal.pntd.0000632 20300513 PMC2838779

[B38] O'Neill SL Gooding RH Aksoy S Phylogenetically distant symbiotic microorganisms reside in Glossina midgut and ovary tissues Medical and Veterinary Entomology 1993 7 377 383 8268495 10.1111/j.1365-2915.1993.tb00709.x

[B39] Hurst GDD Jiggins FM Problems with mitochondrial DNA as a marker in population, phylogeographic and phylogenetic studies: the effects of inherited symbionts Proc R Soc 2005 B 272 1525 1534 10.1098/rspb.2005.3056 PMC1559843 16048766

