# Supplementary material for: Temporal stability of Glossina fuscipes fuscipes populations in Uganda
Source: Parasit Vectors. 2011 Feb 14;4:19. doi: 10.1186/1756-3305-4-19 (PMC3045980; doi:10.1186/1756-3305-4-19)
Supplement: Additional file 1 — Table S1. FIS values for the 16 microsatellite loci. Significance was assessed at p < 0.05 (*) and a Bonferroni-corrected value of p < 0.0028 (bold). Low variability precluded calculation of FIS in some populations (n/a). [file 1756-3305-4-19-S1.DOC]

Table S1: F_IS_ values for the 16 microsatellite loci. Significance was assessed at p < 0.05 (*) and a Bonferroni-corrected value of p < 0.0028 (bold). Low variability precluded calculation of F_IS_ in some populations (n/a).

| Samples | Microsatellite locus | | | | | | | | | | | | | | | |
| --- | --- | --- | --- | --- | --- | --- | --- | --- | --- | --- | --- | --- | --- | --- | --- | --- |
|  | A03b | B05 | B20b | CAG29 | C5b | C7b | D101 | GpCAG133 | GpC10 | Pgp28 | Gmm8 | GmmA06 | GmmB20 | GmmD15 | GmmL03 | GmmL11 |
| BN - 0 | 0.206* | -0.071 | 0.246 | -0.127 | n/a | 0.313 | 0.231 | 0.040 | 0.035 | 0.123 | 0.028 | 0.005 | 0.090 | 0.137 | -0.157 | 0.067 |
| BN - 8 | 0.248* | 0.078 | 0.190 | n/a | n/a | 0.041 | -0.054 | 0.076 | 0.171 | -0.044 | 0.062 | 0.017 | 0.087 | 0.195 | -0.086 | -0.025 |
| BN - 12 | -0.005 | 0.074* | -0.059 | -0.050 | n/a | 0.012 | -0.021 | -0.070 | 0.084 | **0.320*** | -0.126* | 0.057 | 0.107 | 0.165 | -0.053 | 0.070 |
| BU - 0 | 0.185 | -0.076 | 0.132* | n/a | n/a | 0.076 | 0.145 | -0.104 | 0.012 | -0.044 | 0.050 | 0.211 | -0.032 | n/a | -0.148 | 0.139 |
| BU - 8 | -0.020 | 0.062 | 0.019 | 0.483 | n/a | 0.115 | -0.031 | 0.129 | 0.274 | -0.084 | 0.235 | -0.080 | -0.096 | -0.056 | -0.158 | -0.024 |
| BU - 12 | 0.082 | 0.204 | 0.087* | n/a | n/a | -0.125 | -0.036 | -0.307 | 0.433* | 0.012 | -0.058 | -0.182 | 0.024 | -0.056 | -0.016 | 0.171 |
| JN - 0 | 0.018 | 0.158 | -0.084 | n/a | n/a | -0.036 | -0.054 | -0.013 | 0.112 | 0.005 | -0.018 | 0.089 | -0.053 | 0.280 | -0.073 | 0.079 |
| JN - 13 | -0.083 | -0.150 | 0.219 | n/a | n/a | -0.178 | 0.093 | 0.199 | 0.286 | 0.118 | n/a | 0.121 | -0.065 | -0.120 | -0.214 | 0.165 |
| MK - 0 | 0.024 | -0.001 | 0.062 | 0.009 | n/a | 0.032 | n/a | -0.175 | -0.059 | -0.052 | 0.016 | -0.025 | -0.090 | -0.189 | -0.077 | -0.262 |
| MK - 8 | -0.009 | 0.105 | 0.203 | -0.211 | n/a | -0.057 | n/a | -0.131 | -0.066 | -0.387 | -0.070 | -0.181 | 0.132 | -0.126 | -0.158 | 0.097 |
| MK - 12 | -0.033 | -0.079 | 0.043 | -0.235 | n/a | 0.250* | n/a | 0.109 | 0.051 | 0.172 | -0.077 | -0.043 | **0.580*** | 0.073 | 0.062 | -0.121 |
| MS - 0 | 0.068 | -0.161 | 0.006 | -0.052 | -0.081 | 0.176 | 0.037 | -0.068 | 0.020 | -0.021 | -0.158 | -0.101 | -0.169 | 0.009 | -0.180 | -0.082* |
| MS - 13 | 0.054 | -0.028 | 0.145 | -0.053 | 0.148 | 0.106 | -0.037 | 0.394* | -0.250 | 0.122 | -0.134 | -0.108 | -0.022 | 0.458 | -0.010 | **0.287*** |
| OK - 0 | 0.044 | 0.130 | 0.048 | -0.007 | n/a | 0.429* | 0.298 | -0.007 | -0.116 | 0.145 | -0.011 | -0.013 | 0.074 | -0.027 | 0.109 | 0.379* |
| OK - 8 | 0.017 | 0.080 | -0.053 | n/a | n/a | -0.094 | 0.101 | -0.021 | 0.003 | -0.071 | -0.230 | -0.027 | 0.065 | -0.018 | 0.021 | -0.170 |
| OK - 12 | 0.032 | 0.010 | 0.096 | n/a | n/a | -0.108 | -0.030 | -0.110 | -0.051 | 0.290 | -0.145 | 0.128 | -0.241 | -0.041 | -0.034 | 0.152 |
| OT - 0 | 0.024 | 0.224 | -0.026 | 0.164 | 0.122 | 0.079 | -0.030 | 0.040 | -0.011 | 0.087 | 0.093 | 0.076 | -0.100 | 0.189 | -0.024 | -0.030 |
| OT - 11 | -0.040 | -0.065 | 0.057 | -0.102 | n/a | -0.119 | 0.136 | 0.048 | -0.087 | **0.285*** | 0.489* | -0.068 | 0.032 | 0.054 | 0.055 | -0.018 |
