# Supplementary material for: Temporal stability of Glossina fuscipes fuscipes populations in Uganda
Source: Parasit Vectors. 2011 Feb 14;4:19. doi: 10.1186/1756-3305-4-19 (PMC3045980; doi:10.1186/1756-3305-4-19)
Supplement: Additional file 2 — Table S2. Pairwise estimates of genetic differentiation (Jost's DEST) between samples taken from seven populations of G. f. fuscipes. Estimates of differentiation (below diagonal) and associated standard error (above diagonal) between populations of flies sampled at the same site but different times are shaded in grey. [file 1756-3305-4-19-S2.DOC]

Table S2: Pairwise estimates of genetic differentiation (Jost’s D_EST_) between samples taken from seven populations of *G. f. fuscipes*. Estimates of differentiation (below diagonal) and associated standard error (above diagonal) between populations of flies sampled at the same site but different times are shaded in grey.

|  | BN  0 | BN  8 | BN  12 | BU  0 | BU  8 | BU  12 | JN  0 | JN  13 | MK  0 | MK  8 | MK  12 | MS  0 | MS  13 | OK  0 | OK  8 | OK  12 | OT  0 | OT  11 |
| --- | --- | --- | --- | --- | --- | --- | --- | --- | --- | --- | --- | --- | --- | --- | --- | --- | --- | --- |
| BN  0 |  | 0.005 | 0.004 | 0.051 | 0.047 | 0.050 | 0.057 | 0.056 | 0.025 | 0.027 | 0.027 | 0.048 | 0.052 | 0.043 | 0.044 | 0.050 | 0.034 | 0.040 |
| BN  8 | -0.005 |  | 0.003 | 0.058 | 0.053 | 0.058 | 0.064 | 0.060 | 0.022 | 0.027 | 0.025 | 0.059 | 0.057 | 0.052 | 0.054 | 0.059 | 0.033 | 0.042 |
| BN 12 | -0.001 | 0.002 |  | 0.057 | 0.055 | 0.058 | 0.064 | 0.062 | 0.023 | 0.025 | 0.024 | 0.057 | 0.058 | 0.051 | 0.051 | 0.055 | 0.041 | 0.048 |
| BU  0 | 0.282 | 0.271 | 0.289 |  | 0.005 | 0.004 | 0.031 | 0.044 | 0.083 | 0.088 | 0.086 | 0.072 | 0.068 | 0.017 | 0.019 | 0.016 | 0.078 | 0.081 |
| BU  8 | 0.273 | 0.266 | 0.286 | 0.004 |  | 0.003 | 0.033 | 0.045 | 0.081 | 0.086 | 0.085 | 0.069 | 0.070 | 0.011 | 0.010 | 0.012 | 0.073 | 0.078 |
| BU 12 | 0.300 | 0.293 | 0.314 | 0.001 | -0.002 |  | 0.031 | 0.041 | 0.083 | 0.087 | 0.085 | 0.074 | 0.072 | 0.012 | 0.012 | 0.012 | 0.081 | 0.084 |
| JN  0 | 0.337 | 0.339 | 0.362 | 0.122 | 0.114 | 0.121 |  | 0.005 | 0.080 | 0.084 | 0.081 | 0.066 | 0.060 | 0.037 | 0.040 | 0.036 | 0.080 | 0.079 |
| JN  13 | 0.344 | 0.344 | 0.373 | 0.141 | 0.125 | 0.135 | -0.003 |  | 0.079 | 0.082 | 0.078 | 0.072 | 0.072 | 0.046 | 0.048 | 0.044 | 0.078 | 0.078 |
| MK  0 | 0.088 | 0.096 | 0.094 | 0.478 | 0.476 | 0.497 | 0.523 | 0.534 |  | 0.006 | 0.007 | 0.077 | 0.072 | 0.073 | 0.077 | 0.079 | 0.036 | 0.036 |
| MK  8 | 0.110 | 0.109 | 0.108 | 0.484 | 0.483 | 0.505 | 0.523 | 0.536 | 0.005 |  | 0.006 | 0.079 | 0.073 | 0.078 | 0.079 | 0.080 | 0.048 | 0.042 |
| MK  12 | 0.108 | 0.118 | 0.103 | 0.470 | 0.469 | 0.488 | 0.518 | 0.535 | 0.002 | -0.003 |  | 0.077 | 0.072 | 0.074 | 0.077 | 0.079 | 0.042 | 0.040 |
| MS  0 | 0.286 | 0.317 | 0.329 | 0.420 | 0.407 | 0.437 | 0.405 | 0.380 | 0.458 | 0.451 | 0.463 |  | 0.022 | 0.066 | 0.067 | 0.065 | 0.063 | 0.071 |
| MS 13 | 0.285 | 0.316 | 0.325 | 0.374 | 0.363 | 0.392 | 0.330 | 0.309 | 0.480 | 0.469 | 0.478 | 0.019 |  | 0.065 | 0.065 | 0.066 | 0.061 | 0.069 |
| OK  0 | 0.282 | 0.279 | 0.301 | 0.048 | 0.028 | 0.033 | 0.119 | 0.116 | 0.499 | 0.513 | 0.500 | 0.357 | 0.324 |  | 0.003 | 0.004 | 0.067 | 0.071 |
| OK 8 | 0.265 | 0.264 | 0.277 | 0.045 | 0.027 | 0.032 | 0.124 | 0.122 | 0.485 | 0.501 | 0.489 | 0.355 | 0.319 | -0.007 |  | 0.002 | 0.069 | 0.073 |
| OK 12 | 0.263 | 0.264 | 0.279 | 0.047 | 0.034 | 0.036 | 0.126 | 0.120 | 0.477 | 0.491 | 0.481 | 0.343 | 0.315 | -0.005 | -0.007 |  | 0.069 | 0.073 |
| OT 0 | 0.127 | 0.133 | 0.159 | 0.419 | 0.409 | 0.438 | 0.455 | 0.438 | 0.163 | 0.177 | 0.187 | 0.372 | 0.408 | 0.403 | 0.406 | 0.398 |  | 0.007 |
| OT 11 | 0.115 | 0.126 | 0.152 | 0.422 | 0.414 | 0.438 | 0.468 | 0.454 | 0.151 | 0.166 | 0.177 | 0.374 | 0.399 | 0.412 | 0.415 | 0.399 | 0.013 |  |
